# Urethral Chancroid, Cicatricial Stricture

**Published:** 1880-08

**Authors:** Henry P. Cooke

**Affiliations:** Assistant Surgeon; U. S. Marine Hospital Service


### Clinical Reports.

---

### URETHRAL CHANCROID, CICATRICIAL STRICTURE.

REPORTED BY HENRY P. COOKE, M. D., ASSISTANT SURGEON U. S. MARINE HOSPITAL SERVICE.

F., SEAMAN, aged 27 years, placed under treatment January 9, 1880, for chancroid of penis, and a discharge from the urethra. On examination a small lump was found in the urethra about 1¾ inches from the meatus, and it was believed that the discharge arose from a chancroid located at this point within the canal. January 24th, patient complained of symptoms of stricture of the urethra, which was found to be located in the seat of chancroid above described. The chancroid having healed, treatment for the stricture was instituted by the method of gradual dilatation, which was continued until a No. 6 sound could be passed. Beyond this point the use of sound produced no effect, and another chancroid appearing on the glans penis, treatment was suspended until it should have healed. From latter part of February to April 20th, patient was lost sight of. At the latter date he again appeared with chancroid still existing, having assumed the serpiginous type, and for this he was treated until June 27, when it finally healed. Examination now showed a tight stricture, through which it was impossible to introduce a No. 1 catheter. By the use of filiforn bougie, small silver probes, and finally catheter, dilatation was carried to the extent of admitting a No. 1 steel sound, and from this it was gradually extended until No. 3 could be introduced. Beyond this it was found impossible to proceed, and divulsion, with Thompson instrument, was attempted, patient being under influence of ether.

The instrument was turned until the index marked about No. 4, when with a sudden snap, it broke, the shoulder against which the point of the lever rests having been pried from its bed in the groove of the shaft. Subsequent to this two separate attempts were made with the same results, although the instrument was repaired very thoroughly after each accident. As the stricture was too tight to admit Maissoneuve's urethrotome, it was decided to attempt to incise it from within, with an ordinary tenotome; and to this end the patient was etherized, a grooved director introduced, and a long-bladed tenotome made to follow the groove until the stricture was reached. With the knife inserted up to the shoulder of the handle, the stricture could be touched with only a small part of the cutting edge, and with this it was nicked until a No. 4 sound could be passed. Maissonneuve's instrument was now introduced, and the blades separated until the index marked 40, when the knife of the instrument was drawn through the stricture, and the urethrotome removed. A No. 12 sound was immediately passed, and the treatment by dilatation commenced. The resistance of the stricture was shown by the fact that the lower blade of the urethrotome, a powerful instrument, was bent several lines out of position; and when the knife passed through the stricture the sound was that produced by cutting hard rubber cartilage.

